# Simulation of future global warming scenarios in rice paddies with an open-field warming facility

**DOI:** 10.1186/1746-4811-7-41

**Published:** 2011-12-06

**Authors:** Muhammad Ishaq Asif Rehmani, Jingqi Zhang, Ganghua Li, Syed Tahir Ata-Ul-Karim, Shaohua Wang, Bruce A Kimball, Chuan Yan, Zhenghui Liu, Yanfeng Ding

**Affiliations:** 1 College of Agriculture, Nanjing Agricultural University, Nanjing 210095, Jiangsu, P.R. China; 2 Key Laboratory of Crop Physiology and Ecology in Southern China, Ministry of Agriculture, Nanjing 210095, Jiangsu, P.R. China; 3 US Arid Land Agricultural Research Center, USDA, Agricultural Research Service, 21881 North Cardon Lane, Maricopa, AZ 85238, USA; 4 Institute of Crop and Nuclear Technology Utilization, Zhejiang Academy of Agricultural Sciences, Hangzhou 310021, P.R. China

**Keywords:** Ecosystem warming, climate change, canopy temperature, global change, infrared heating, plant-climate interactions, rice, Yangtze River valley

## Abstract

To simulate expected future global warming, hexagonal arrays of infrared heaters have previously been used to warm open-field canopies of upland crops such as wheat. Through the use of concrete-anchored posts, improved software, overhead wires, extensive grounding, and monitoring with a thermal camera, the technology was safely and reliably extended to paddy rice fields. The system maintained canopy temperature increases within 0.5°C of daytime and nighttime set-point differences of 1.3 and 2.7°C 67% of the time.

## Background

Rice (*Oryza sativa *L.) is a staple food for more than 3 billion humans, mainly in Asia. Its demand will increase because the world's population is expected to reach 9.1 billion by year 2050, including 5.5 billion in Asian countries [1]. Despite a high degree of yield increase, mainly due to Green Revolution technologies, average rice yields show a high magnitude of instability depending on weather and monsoon anomalies [2]. Climate change in addition to high population growth is pressuring the thin margin between supply and demand of rice [3]. Most of our insights regarding impacts of climate change consider observed and predicted changes in temperature over the next century [4]. By the end of this century, global surface air temperature is expected to have increased by 1.1-6.4°C relative to the average temperature during 1980-1999. The best estimate for the expected air temperature increase for Intergovernmental Panel on Climate Change (IPCC) low population growth scenario (B1) is 1.8°C with likely range of 1.1°C to 2.9°C, while the best estimate for a high growth scenario (A2) is 3.4°C with likely range of 2.0 to 5.4°C [5].

There is substantial spatiotemporal, seasonal, and inter-annual variability in the warming trend. A faster increase in nighttime temperature than daytime temperature is reported for Jiangsu province [6]. Most of the studies conducted to investigate the effect of temperature on different aspects of plants are based on diel mean air temperature, assuming there is no differential influence of nighttime and daytime temperatures [7]. However, the negative impact of high nighttime temperature on rice production is greater than that of daytime or daily mean temperatures [8]. Peng *et al*., found a 10% decrease in rice grain yield for each 1°C rise in nighttime minimum temperature, while yield was not significantly affected by a rise in daytime maximum temperature [7]. Recently, a trend of increasing differential between daytime and nighttime temperatures has been observed in the literature, with more focus on higher nighttime temperature [9-12].

Substantial efforts have been made to simulate the effects of predicted global warming on rice through a variety of closed or partially open warming facilities based on chambers of various designs. However, there are several chamber artifacts which can alter experimental and/or environmental parameters for plants grown in such "closed or partially closed" or controlled environment facilities [13-15]. These parameters are either impossible or difficult to control, depending upon their own gradient and interaction with each others. They include light quantity and quality, wind speed, relative humidity, long-wave radiation, evapotranspiration rates, and CO_2 _concentration. Often growth mediums (soil nutrient status, pot/container size) provided in the controlled environment systems rarely match with field conditions [14]. Consequently abnormal root growth, development, and functioning may result, and they can alter plant size and morphology as well compared to those grown in the field [14]. Open-top chambers (OTCs) have been frequently used to study the effects of different temperature regimes on rice [16-18]. The maximum temperature differences using this technique occur during full sunlight conditions. Although OTCs can heat both air and soil, sunny days are necessary for the heating, and the majority of warming is achieved only during daytime [19].

A promising alternative to warming with chambers and their associated artifacts is the use of infrared heaters (IRH) over open-field plots. The use of IRH to study the response of ecosystems to global warming was started in mid 1990s [20-23]. The temperature rise of a rice canopy through IRH warming is essentially the same as the warming provided by radiant heating from the sun and sky because it directly heats the canopy. The air in and above the canopy is subsequently warmed by convective sensible heat exchange with the canopy (and cooled by latent heat exchange). If the "constant temperature rise" mode of operation is used, as we did herein, the warming by IRH can be directly related to degree of canopy warming expected through global warming [13,24,25].

The amount of energy required to achieve a specified increase in canopy temperature by IRH is influenced by canopy conductance in response to soil moisture conditions, light intensity, temperature, humidity, and wind speed [26]. Less energy is required when the stomates are closed, such as occurs under water stress or at night. However, with warmer leaves, higher vapor pressure occurs in the sub-stomatal cavities in the infrared-warmed canopies, which can create unrealistic vapor pressure gradients between the inside of the leaves and air [25,26]. This problem can be minimized through the introduction of supplemental irrigation for upland crops [13,24,26]. However, it is not required for paddy fields, which are already grown under flooded conditions, so depletion of soil moisture was not an issue in the results reported herein.

Selection of the infrared heaters IRH is important so that thermal radiant properties are suitable for the plant warming application i.e., no significant radiation less than 850 nm and high emissivity [26]. Since most of the commercially available IRH are manufactured for indoor use only, measures are required to make them water-proof and safe for reliable outdoor use. In addition, the heaters need to be deployed in configurations that provide uniform distribution of the warming across the field plots. Kimball *et al*. (2008) arranged infrared heaters in hexagonal fashion and achieved highly repeatable and uniform distribution of warming on the canopy of wheat and Sudan grass [27]. This updated model of IRH had few ecosystem limitations and little disturbance in ecosystem [19]. Rice has additional challenges to adopt such a technique, as it is grown in standing water, and numerous high capacity electrical cables are hazards.

A facility is required to provide warming of field plots to fully assess the likely effects of different scenarios of global warming on future rice production. Infrared heater arrays with the modifications introduced in this study can safely produce open-field warming of rice canopy temperatures like those expected with climate change scenarios in the paddy fields. Our goal was to construct such an open-field warming facility for paddy fields like those in the Lower Reaches of the Yangtze River Valley (the oldest niche of rice production) and elsewhere to simulate differential daytime and nighttime warming under low (B1) and high (A2) population growth scenarios. Danyang FATE (Free-air Temperature Enhancement) facility will help us to predict the fate of rice production in its oldest niche, i.e., the Yangtze River valley.

## Result and discussion

### Experiment I

#### Infrared Heater (IRH) description

**Figure 1:**
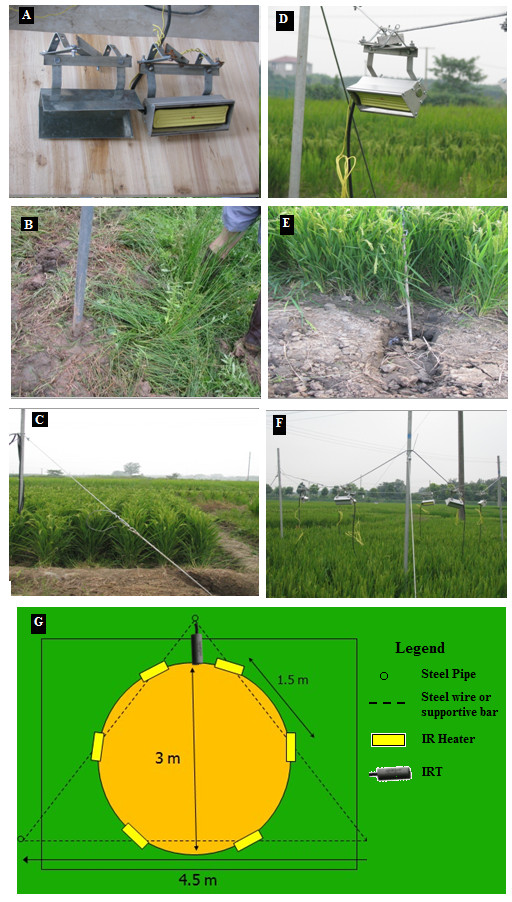
**A, Infrared heater with reflector assembly and dummy heater arrangement used in Exp-I at Jiangning Experimental Station, Nanjing, infrared heater array for rice crop in summer 2008; B, Vertical steel post fixed in the soil; C and E, Supportive cable fixed outside the experimental plot; D, Hanging IR heater on the steel cable; F, Preliminary infrared heater array with infrared heaters; G, Geometric distribution of Infrared heaters used in Exp-I and II**.

Infrared heaters (IRH) [Model FTE-1000 (1000 W, 240 V, 245 mm long × 60 mm wide)] were fitted in reflective housings [Model ALEX-F (254 mm long × 98 mm wide ×89.4 mm high)] manufactured by Mor Electric Heating Association Inc. (Comstock Park, MI, USA) (Figure 1A). These ceramic IRHs emit radiation from their glaze surface with claimed emissivity of 0.96. Due to the manufacturer's restriction (indoor use only) and openings in the housing, there was an electrical hazard due to water intrusion under open-field conditions [27]. Therefore, we reassembled the heater elements and housings, carefully blocking all openings, and all the joints were sealed [Red RTV Silicon Gasket Maker (Oxforce, China)]. The sealant is high temperature and water (and freeze) resistant, with an operational temperature up to 360°C. Although the heater elements can exceed 700°C, many parts of the housing are cooler [27]. The sealant proved to be adequate to protect the IRHs from water over several months (used for two seasons a year, for rice and wheat for three years) at the Danyang FATE site, where rainfall in summer and snowfall in winter are frequent. The heaters proved to be durable without malfunctioning (13 FATE arrays with 78 heaters were used for at least two rice and wheat seasons each, from summer 2008 to summer 2011).

#### Infrared Heater Array

Three steel posts, each of 3 m length, were inserted vertically into the paddy soil to a depth of 0.5 m, making an equilateral triangle of each side 450 cm (Figure 1B, G). A suspension cable (1/8 inch steel cable) was fixed around the posts (Figure 1D, F). Each post was supported by another diagonal steel cable, tied to a 120 cm long stake in the soil (Figure 1C, E, F). At grain filling stage, six IRHs were hung from the suspension cable at a height of 120 cm above the top of the canopy (0.8 times the radius of array), covering a 3-m diameter circular soil area (7.1 m^2^) to from a hexagonal FATE array (Figure 1G). IRHs were tilted at 45° from horizontal and ± 30° from the cable axes to provide uniform distribution of infrared radiation in the plot area (Figure 1D). Each array was equipped with six IRHs (total capacity 6000 W), infrared thermometer (IRT), dimmer, and an electrical distribution unit protected in a weather resistant enclosure (Model ENC12/14, Campbell Sci. Inc., Logan, UT, USA).

A "dummy" array of same size, shape and structure, except with dummy heaters (with the same color of reflecting housing), was also erected (Figure 1A). Both IRH and dummy arrays were deployed in the center of square plots (6 m × 6 m), allowing a buffer strip of at least 6 m at their perimeter. The shading of six IRH from nadir was about 2%. However, because the heaters were deployed around the perimeter of the circular plots, only half of the IRH would shade the plots at any specific time, so the amount of shading over the plots was about 1% [27].

#### Control system

**Figure 2:**
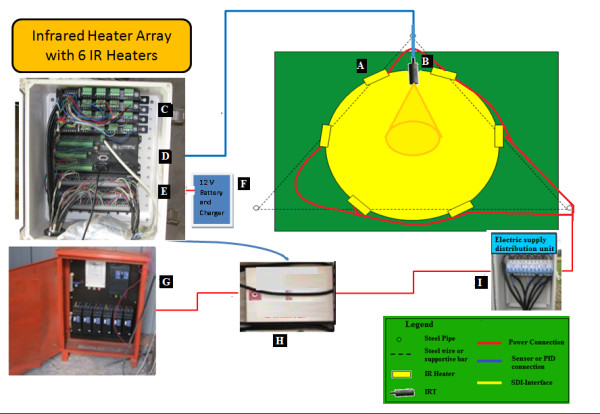
**Functional diagram of an infrared heater array with six heaters deployed over a 3-m-diameter (7.01 m^2^) plot of rice used in Exp-I and II**. A, Infrared heaters (IRH); B, Infrared temperature sensor (IRT); C, current/voltage output modules (SDM-CV04); D, datalogger (CR1000); E, electric supply unit; F, Dimmer; G, electric supply distribution unit.

Rice canopy temperatures in each plot were measured (per second) using infrared thermometers (IRTs; Model SI-121, Apogee Instruments, Inc., Logan, UT, USA) fixed on the southern posts of the triangles at a height of 0.8 m (above plant canopies) pointed north and downward with an angle of 45° from nadir to effectively monitor canopy temperatures of the plots (7 m^2^) (Figure 2). These IRTs were 62 mm long × 23 mm diameter with a field of view (FOV) of 18° and a waveband of 8-14 μm, which corresponds to the atmospheric window so as to minimize reflected sky radiation. These IRTs had a claimed accuracy of ± 0.5°C and a repeatability and uniformity of 0.1°C over a wide range of temperature (-55 to 80°C) and relative humidity (0-100% non-conducive). A datalogger (Model CR1000, Campbell Sci., Inc., Logan, UT, USA) (Figure 2D) equipped with current/voltage output module (Model SDM-CV04, Campbell Sci., Inc., Logan, UT, USA) (Figure 2C) was used to measure signals from the IRTs of reference and heated plots and then compute rice canopy temperatures that were corrected for radiation from the heaters that was reflected from the rice canopies. Then, using a proportional-integrative derivative (PID) control algorithm, the datalogger transmitted 0-10 V signals to dimmers (Model LCED-2484, 240 V, 35 A, 60 Hz, 8.4 KW, Kalglo Electronics Inc. PA, USA) (Figure 2H) to regulate the electric supply to the IRHs.

Using a PID controller subroutine obtained from Campbell Scientific (Campbell Sci., Inc., Logan, UT, USA), Kimball (2005) developed a program to control infrared heaters using the components listed in the previous paragraph [26]. This program was modified to suit the configuration of our particular heating system and warming treatments (for Exp. I, II & III). Scaling of the PID signals was introduced for smoother control of the IRHs so that there was less oscillation and acting like an ON/OFF controller compared to the original program. A car battery with charger was used as a source of 12 V power for the datalogger (Figure 2D, F). The set point differences between the heated and the corresponding reference plots were 1.3°C during daytime and 2.7°C during nighttime to achieve warming averages resembling the lower and upper limits of the B1 scenario of global warming predicted by IPCC (Table SPM.3, page 13) [5].

### Experiment II

#### Construction of Danyang-FATE facility

**Figure 3:**
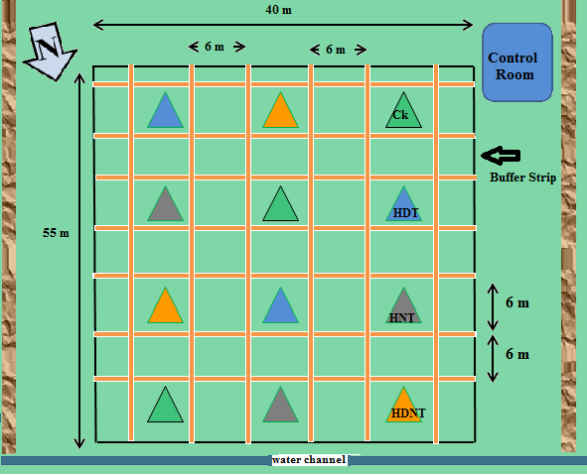
**Plot plan for Danyang-FATE facility with 12 plots for Exp. II**. Experimental plots (6 m × 6 m) separated by buffer strip (6 m × 6 m) and alleyways (0.5 m). Ck; control (ambient temperature); HDT, high daytime temperature; HNT, high nighttime temperature; HDNT, high daytime and nighttime temperature.

For Experiment II (Exp-II) in summer 2009, we established the Danyang FATE facility at Danyang, Jiangsu, China (119°27' E, 31°54 N). We constructed 18 FATE arrays within a 90 m × 60 m area. No array was within 6 m of any other array in order to create a buffer strip so that heated plots did not also warm reference plots (Figure 3, only 12 arrays visible in this figure). Exp-II was conducted with three replications (Figure 3) and with two yearly repetitions (2009 and 2010).

**Figure 4:**
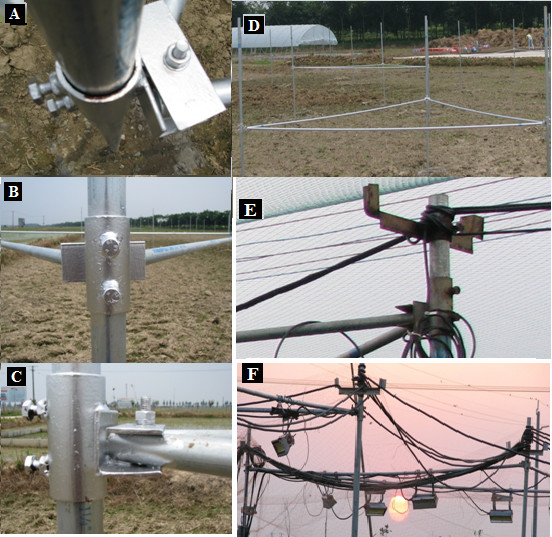
**Description of fittings for modified infrared heater array (Exp-II and III) (A, B, and C) Different views of steel angle attached on a coupling**. Vertical pipe was inserted in coupling and was tightened with two screws on the coupling. Horizontal pipes were tightened with the help of a flat steel plate, nut and bolt. (D). Overall view of triangular pipe system to deploy the infrared heaters. (E) T shaped hanger to carry cable above ground. (F) distribution of cables above a FATE array.

**Figure 5:**
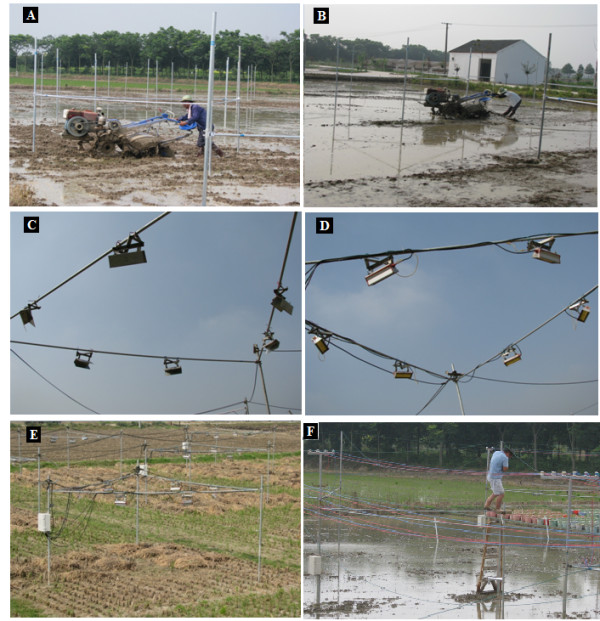
**A-B: Soil preparation for rice transplanting, C-D: bottom views of dummy and infrared heater arrays, E: aerial views of FATE site after harvesting rice, F: installation of new cables above the IRH arrays before rice transplantation (2010)**. Structural stability of FATE array is demonstrated by the weight of a man on the supportive bars of the FATE array during installing of the new electric cables.

**Figure 6:**
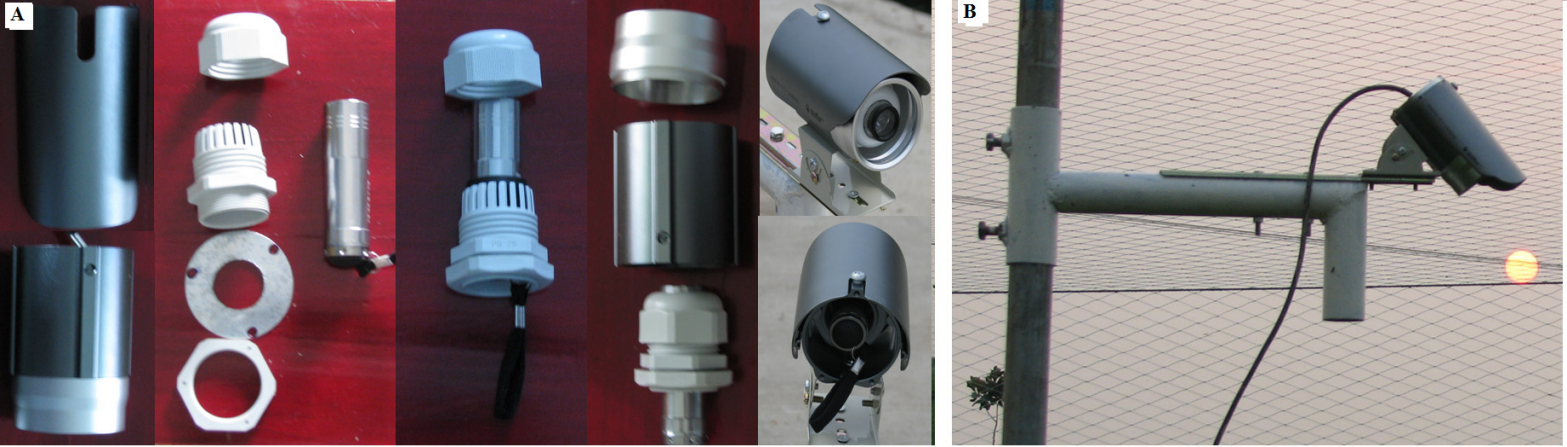
**Solar radiation shield for Apogee SI-121 infrared temperature sensor**. A. A. Fitting of infrared thermometer inside a closed circuit television camera cover B. Infrared tthermometer fixed on an extended arm attached with a coupling; vertical pipe was inserted in coupling and tightened with the help of screws.

On 10^th ^June 2009 (18 days before rice transplantation), supporting posts were fixed in the soil by adding concrete to a depth of 60 cm (Figure 4A). To restrict movement of the concrete, we used wood shuttering covered with plastic sheets (which were removed before soil preparation). Instead of suspension cables, we used steel bars attached to the vertical posts using steel couplings (Figure 4 A, B, C). IRHs or dummy heaters were attached to the horizontal bars to make hexagonal shapes, as in Exp-I (Figure 5C, D, E). For all the plots, the IRTs were deployed on an arm from the south posts at the same height (0.8 m above the rice canopy) and angle (45° from nadir), oriented towards north (from southern edge of plot) and pointed at the centers of the plots (Figure 6B). The IRTs were mounted inside closed circuit television camera covers (Figure 6A) to provide solar radiation shields which reduced the temperatures of the sensor bodies during daytime and helped to assure that the temperatures were uniform within the bodies of the IRTs.

**Figure 7:**
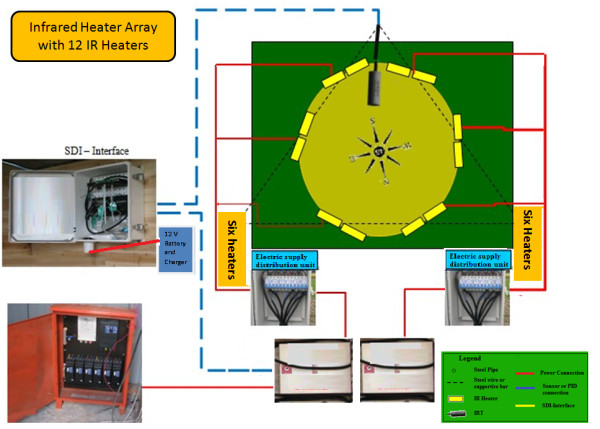
**Functional diagram of infrared heater array with 12 heaters deployed over 3-m-diameter (7.01 m^2^) rice plot**. A: Infrared heaters, B: Infrared thermometer, C: current/voltage output modules (SDM-CV04), D: datalogger (CR1000), E: electric supply control unit, F: dimmer, G: electric supply distribution unit.

**Figure 8:**
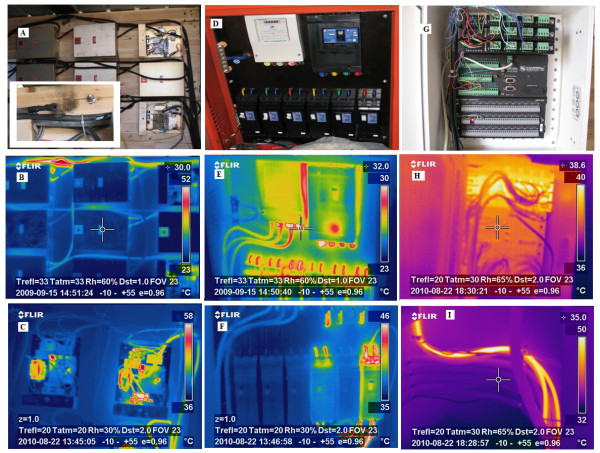
**Control room safety checked through periodic thermographs of electric equipments and cables**. A: picture of burning electric supply cable, B: thermograph showing increased temperature of cable before burning. C: thermograph of non-working and working dimmer, D-E: picture and thermograph of main electrical panel, F: thermograph of power supply to heater arrays. G-H: picture and thermograph of data control unit, consisting of current/voltage output modules (SDM-CV04), datalogger (CR1000) and multiplexer (AM 16/32), I: thermograph of power supply to IRH array with 12 heaters.

A control room was built (at the western periphery of the FATE site) (Figure 3), where one datalogger, one multiplexer, four current/voltage output modules [arranged in a weather resistant enclosure (Model ENC12/14, Campbell Sci. Inc., Logan, UT, USA) (Figure 7, 8G], 15 dimmers (Fixed on the wooden walls of control room, Figure 8A), and one DC battery were housed. For each heated array, one IRT and a dimmer were used, and each check or reference plot also had an IRT. Four current/voltage output modules (SDM-CV04) and one multiplexer (AM16/32, Campbell Sci. Inc. UT, USA) were connected to datalogger in order to measure all the canopy temperatures and to regulate heating of all the arrays (Figure 2C, D, E). A reliable electrical power supply was assured through a 30 kVA (kilovolt-amperes) transformer to the control room from the main power lines.

T-shaped steel hangers were attached to the top of a vertical post of each array to carry electrical cables and avoid any contact of the cables with irrigation water (Figure 4E). Electrical distribution units with control switches for each array were secured in weather resistant enclosures (ENC 12/14, from Campbell Sci. Inc. UT, USA) and attached to one of the posts in each array (Figure 2I). Electrical power from the control room was connected to the electrical supply units for each IRH array, which then divided and supplied electricity to all six heaters of each array (Figure 2).

Warming treatments were imposed between August 31 and October 14 (2009) and September 2 and October 13 (2010). The set point differences between the heated and corresponding reference plots were the same as described in Exp-I (1.3°C during daytime and 2.7°C during nighttime) using the same equipment as described for Exp-I, but with modifications as described above. However, two additional treatments, HNT (High Nighttime Temperature, + 2.7°C CK) and HDT (High Daytime Temperature, +1.3°C CK) were applied in addition to HDNT (High Daytime and Nighttime Temperature, +1.3°C/+2.7°C CK) using the same equipment as described for Exp-I. Each treatment was applied in triplicate (four treatments, three replications, 12 arrays) and averages from two experiment years (2009 and 2010) are used in this paper.

### Experiment III

**Figure 9:**
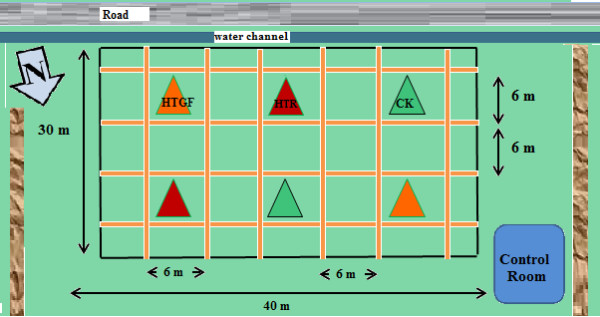
**Plot plan for Exp- III (located at southern side of site of Exp-II)**. Experimental plots (6 m × 6 m) separated by buffer strip (6 m × 6 m) and alleyways (0.5 m). Ck; control (ambient temperature); HTR, high temperature at reproductive stage of rice; HTGF. High temperature at grain filling stage of rice.

Experiment III (Exp-III) was conducted in 2010 during the rice reproductive (August 12 to September 3, 2010) and grain filling stages (September 2 to October 12, 2010) to extend the degree of warming to simulate the A2 scenario. We joined two IRHs through an extended hanger, thus doubling the number of IR heaters per hexagonal array, as described for Exp-II (Figure 7). Four IRTs were fixed on the supportive bars in the south, northeast, north, and northwest sides of heated and reference plots; and their averages were used in the experiment. The height of IRH above the rice canopy of heated plots during reproductive stage was adjusted once, i.e., after 10 days of heating treatment, however, on plots where warming was applied during grain filling stage, it was not required. All IRTs were kept at same height (0.8 m above the top of the rice canopy) and were pointed towards the center of the plot. The set-point difference between heated and corresponding reference plots was kept +2.7°C during daytime and +5.7°C during nighttime to achieve averages resembling upper and lower limits of the A2 scenario of the global warming predicted by IPCC (Table SPM.3, page 13) [5]. Each treatment was applied in duplicate, and their averages are used in this paper (Figure 9).

#### Structural stability

**Figure 10:**
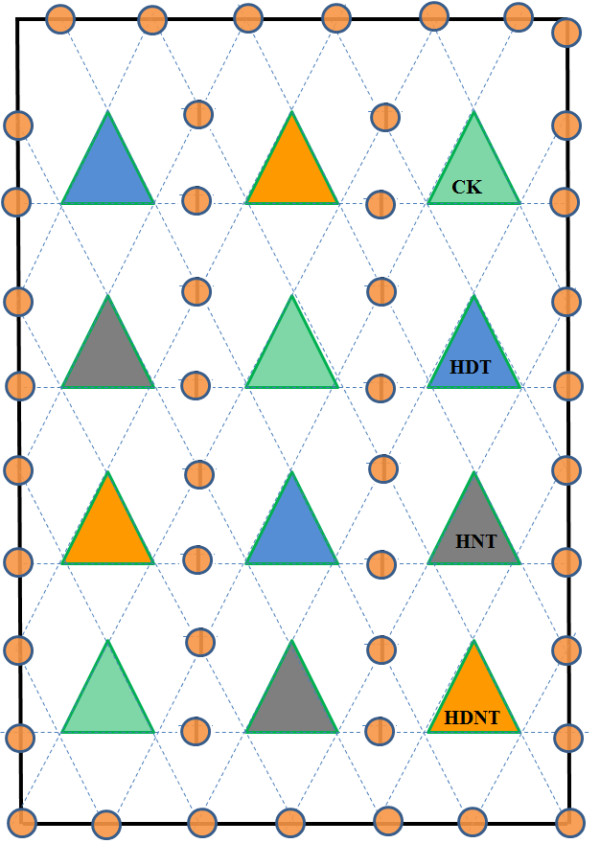
**Sketch of proposed model of hanging cables across the site, with supporting pillars at periphery of FATE site and in buffer strips between FATE arrays**. Ck; control (ambient temperature); HDT, high nighttime temperature; HNT, high nighttime temperature; HDNT, high daytime and nighttime temperature.

In Exp-I, severe problems were initially observed regarding structural stability and safe operation of arrays in rice paddy fields. Paddy soil alone was not able to provide solid support for the posts and heaters. We considered different options, including placing a solid metallic ring around the array, embedding the base of the array in concrete, and hanging cables on the pillars across the FATE site and in the buffer strips of the main experimental area (Figure 10). Placing a metallic ring was ruled out due to problems with cultural practices and the need to study responses from long-term warming at the same location. Hanging cables with supportive pillars can cause problems in height adjustment because the expected heavy load of the heaters would have caused a large difference in the heights of the cables near the centers between pairs of the pillars compared to close to a pillar. Therefore, we decided the best option was anchoring the bases of the vertical posts in concrete. However, a risk of damaging the soil physical and chemical properties from the concrete existed, so, to avoid this problem, wood shuttering and plastic covers were placed around the concrete. The loads of the cables on the posts of arrays were reduced through installation of additional posts with T hangers in the buffer strip between the arrays (Figure 5F). A man standing on the horizontal bars of the FATE array is visible (Figure 5F). These precautions served their purpose well, and achieving stability of the arrays in Exp-I led to successful construction of the FATE site for Exp-II and III.

#### Safety Measures

Strict safety measures were taken to avoid electric hazards at the FATE site (Exp-II and III). All electrical equipment in the control room and the IRHs (each array separately) were connected using 2-mm wire to copper-clad steel (with 1inc diameter and 1.23-m length) grounding rods driven into the soil. In each array, IRHs were tightly fastened to the supporting posts to provide additional grounding. All points of potential electric hazards were regularly checked manually, and thermal images of all electric supply and control units, datalogging equipment, and dimmers were captured using a Thermacam (Figure 8). Differences in surface temperatures of electric cables and dimmers for working and non-working conditions are clear from the thermographs. Burning of an electric cable occurred in 2009 (Figure 8A, B) due to over-heating. All respective cables were immediately replaced with reinforced insulation to avoid this problem again, and at the end of the 2009 experiment, all cables (4-mm thick, braided, 25 A) were replaced with cables of higher electric capacity (8-mm thick, solid). In addition to the T hangers on a vertical post of each array, four posts with T-shaped hangers were anchored in the buffer strip with concrete. Electrical cables from these additional hangers were connected to the electrical supply unit of each array.

#### Data acquisition and thermal images

Like Exp. I and II, temperature signals from the IRTs were measured by the datalogger, and 15-min averages were stored in its database and output for Exp-III as well. The corrections in IRT canopy temperatures (from the heated plots) that were made to adjust for radiation from the IRHs that was reflected from vegetation [13] were doubled to account for the doubled number of heaters. Based on sensed temperatures, signals were transmitted through current/voltage output module (SDM-CV04) to the dimmers, which ultimately regulated the IRHs through regulating power supply to each array, according to the target set-point and treatment to ensure stable warming. For all three experiments described here, daily sunrise and sunset timings were automatically determined for each day by the datalogger through latitude and longitude of experiment site. Stored data was acquired from the datalogger using Loggernet 3 (Campbell Sci. Inc. UT, USA) regularly (either twice or thrice a week) during the experiments. Diel, daytime, nighttime hourly averages of reference and heated plots, and respective temperature differences (ΔT) were calculated from 15-minute averages. Canopy thermal images were taken using a ThermaCAM camera (Model P25, FLIR systems, Boston, USA), as described in our earlier experiments [28]. In 2010, a weather station (WatchDog Model 2700, Spectrum Technologies, East-Plainfield, IL, USA) was installed at FATE site (about 200 m from the arrays) to obtain ancillary weather data, including solar radiation, air temperature, wind speed and direction at 2 m above the soil, rainfall, and relative humidity.

### Performance of infrared heating arrays

#### Simulation of B1 Scenario

**Figure 11:**
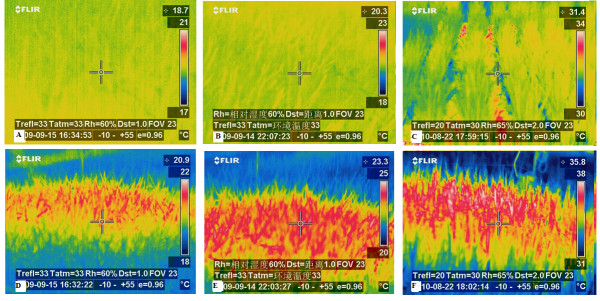
**Thermocam images of rice canopy warmed by six infrared heaters, deployed over 3-m-diameter (7.01 m^2^) rice crop**. A, B and C are thermographs of reference plot (CK) while D, E (HDNT, High daytime and nighttime temperature, reference+1.3/+2.7°C) and F (HT, using 12 infrared heater arrays, Exp-III, +2.7/+5.7°C) for heated plots. A, B, D and E for Exp-II and C and F are for Exp-III.

**Figure 12:**
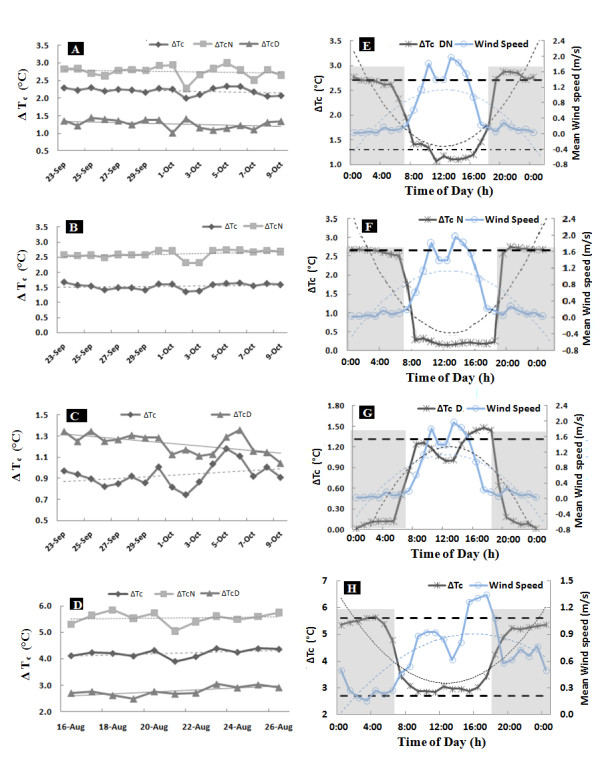
**Performance of infrared heaters on 1.3-m-tall rice crop at Danyang FATE facility, Danyang, China, under different warming treatments**. A-D. Average diel, nighttime and daytime rice canopy temperature increases of the heated plots for 16 days during summer 2010 E-H. Achieved temperature differences (ΔT) and wind speeds (averaged over 23 September and 10 October, 2010) vs. time of day (Time of the nighttime, Beijing Standard Time +8:00 GMT) (Horizontal lines indicate target temperatures).

**Figure 13:**
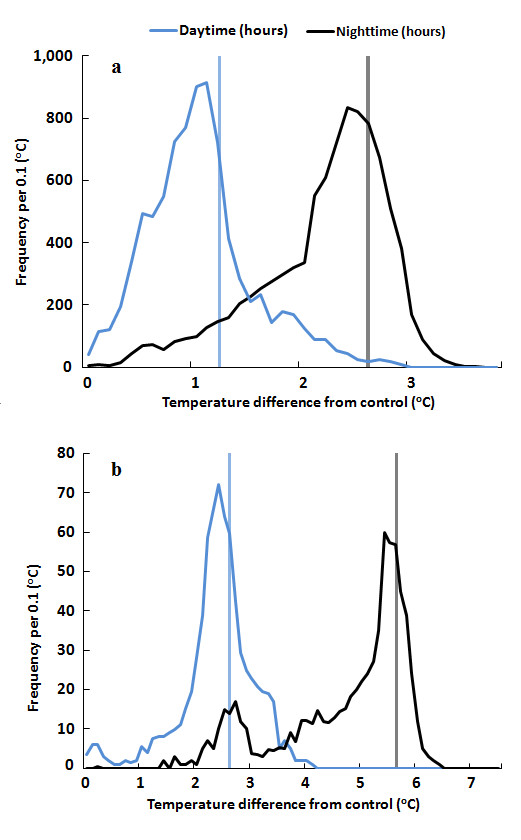
**Frequency distribution of daytime/nighttime temperature differentials between FATE warmed and reference plots measured with IRTs in 2010 (a)**. for Exp-II (1020 h, 2009 and 2010), (b). for Exp III (504 h). Canopy temperature increases in Exp-II (using six infrared heaters per 3-m-diameter array) includes HDNT (High daytime and nighttime temperature, reference+1.3/+2.7°C), HNT (High nighttime temperature, reference+2.7°C) and HDT (High daytime temperature, reference+1.3°C). These treatments were applied between 23 September and 10 October, 2010. In Exp-III, the temperature treatment (HT, using 12 infrared heater arrays, Exp-III, +2.7/+5.7°C) was applied between 16-29 August 2010 (over 504 h).

The IRHs provided uniform warming during daytime (0600-1800 h BST, +08:00 GMT) and nighttime (1800-0600 h BST, +08:00 GMT) following natural diurnal temperature patterns (Figure 11A, B, D, E). Average canopy temperatures (T_c_) were 20.40°C (22.48°C/18.30°C daytime/nighttime) and 21.01°C (22.96°C/19.05°C daytime/nighttime) during 2009 and 2010. Average differences in canopy temperature (ΔT_c_= T_c _of IRH-warmed-T_c _reference plot) under different temperature treatments varied from 0.94°C (daytime, HDT, 2010) to 2.61°C (nighttime, HDNT, 2010). Degrees of achieved warming in Exp-II varied in different treatments and at different temporal scales and were inversely proportional to the prevailing wind speed (U, m s^-1^) (Figure 12E, F, G). The average degree of canopy warming during daytime (ΔT_c D_) and nighttime (ΔT_c N_) varied from 1.27 to 2.75°C, with diel ΔT_c _of 2.21°C, which falls within the range of B1 scenario (1.1-2.9°C). The system was able to track the set-point temperature differences well with 67% and 68% of the observations falling within ± 0.5°C of nighttime (2.7°C) and daytime targets (1.3°C) (Figure 13A). Some of the variability near dawn and dusk was due to transition periods between daytime and nighttime warming, which is in agreement with an earlier report [13,29].

Simple correlation analysis showed that wind speed (U, m s^-1^) had a significant influence on the diurnal cycle of canopy warming (ΔTc) with a correlation coefficient of -0.93 (HDNT). Temporal variability of IRHs and other systems under windy conditions was consistent with previously reported studies [13,26,27,30]. Decreased effectiveness of IRH under high wind velocity reported for rice, under controlled greenhouse conditions cannot perfectly resemble the field conditions, where wind speed greatly varies and has a diurnal pattern [30]. Moreover, Mohammad and Tarpley (2009) had larger IRHs to increase only nighttime temperature, when field conditions generally experience lower wind speed [30]. As expected, maximum warming was achieved during nighttime, when conditions are generally calm and stomata are closed. The diurnal warming patterns that can be achieved with infrared warming systems like presented herein and their relation with wind speed has previously been reported [13,27]. Generally, during some of the time, high wind speeds prevent target levels of warming to be attained. Using larger heaters would increase the percentage of time that target warming can be achieved, but of course electrical power requirements and operating expense would increase. Power requirements could be reduced by using heaters with a larger characteristic dimension, which imparts an increased "radiometric" efficiency [26,31]. Judicious arrangement of heaters in a honeycomb pattern could improve "geometric" efficiency, especially at larger plot scales [31].

#### Simulation of A2 Scenario

Similar to Exp-II, warming effects of IRH on canopy temperature (Tc) in Exp-III was uniformly distributed (Figure 11C, F). Greater warming was achieved during nighttime, which is similar to the historical trend observed for the Jiangsu province, where the FATE site is located (Figure 12D, H) [6]. Daytime and nighttime warming averages were 2.81°C and 5.40°C respectively, with a diel average of 4.12°C. This attained warming closely resembles that expected in the A2 scenario (2.0-5.4°C). The diurnal cycle of ΔTc (canopy temperature between heated and reference plots) was negatively correlated with wind speed (path coefficient of -0.65) (Figure 12H). Daytime temperature differentials were within 0.5°C of the daytime 2.7°C target 68% of the time and nighttime 5.7°C target temperature 55% of the time (Figure 13B). As with Exp-II, some of the variability near dawn and dusk was due to the transition periods between daytime and nighttime warming, which is in agreement with earlier reports [13,29].

## Conclusion

This paper describes the first ever deployment of infrared heaters over rice in open paddy fields in order to simulate the effects of global warming scenarios on rice canopy temperatures. Hexagonal arrays of the infrared heaters in combination with infrared thermometers, dimmers, current/voltage output modules and an automatic control system fulfilled the requirements for an appropriate ecosystem warming system for paddy fields. The system safely provided uniform controlled warming over the plots, and it provided reproducible results under natural open-field conditions of wind and light. Safety features were added including sealing and water-proofing the heater assemblies, setting the support posts in concrete, proper grounding of all electrical equipment, and stringing all electrical cables via overhead supports. This warming system can be used to conduct ecosystem warming experiments for evaluating plant responses to different daytime and/or nighttime temperature increases as predicted by low and high greenhouse-gas emission scenarios. The system was able to track the set-point temperature differences adequately with about 67% of the observations falling within ± 0.5°C of target set-points. However, the efficiency and effectiveness of the IRH heating system was reduced at high wind speed. Using larger and/or more efficient heaters would improve the performance percentage, but electrical power costs would increase. At nighttime, when wind speeds were lower and when stomata were closed, greater warming occurred compared to daytime. These qualities of the FATE facility makes it suitable to simulate global warming scenarios and will be helpful to predict vulnerability of rice specifically in the Yangtze River valley and in general throughout the world.

## Methods

### Experimental facility

The present research was part of a project to construct a FATE facility to study the response of a rice-wheat cropping system to expected global warming in the Lower Reaches of the Yangtze River Valley, China. Three experiments were conducted during the summers of 2008-2010 at Jiangning Experimental Station of Nanjing Agricultural University, Nanjing (118°30 E, 31°50 N) and Danyang FATE facility, Danyang, Jiangsu, China (119°27' E, 31°54 N). Site details are described in our previous studies [28,32]. Experiment-I (Exp-I), was conducted in 2008 at Jiangning experimental station to assess the feasibility of modified IRH arrays, similar to those designed by Kimball *et al*., (2008), for paddy rice fields [28]. Experiment-II (Exp-II) and Experiment III (Exp-III) were conducted at Danyang FATE facility to simulate B1 and A2 emission scenarios. Exp-II was conducted for two years (summer 2009 and 2010), while Exp-III (with 12 heaters per array rather than the original 6) was conducted for one year (summer 2010).

### Crop culture

In Exp-I, japonica rice (*Oryza sativa *L. cv. Zhendao88) was grown in the paddy fields, and for Exp-II and Exp-III, two indica hybrids, Shanyou63 (heat resistant) and Teyou559 (heat susceptible) were used. Sensitivity of these cultivars to high temperature has already been reported and tested in our previous experiments [28,33,34]. These cultivars have similar growth behavior and stature. Each array was divided into two equal parts (north to south), and cultivars were randomly assigned to one half or the other of the arrays. One-month-old seedlings were transplanted in the last week of June each year. Local recommendations for fertilizer and irrigation were followed. Warming treatments were applied at the grain filling stage (Exp-I & II, from August 31 through October 14 in 2009 and September 2 through October 13 in 2010). For Exp-III, they were applied during reproductive (August 12 through September 1, 2010) and grain filling (September 2 through October 13 in 2010) stages.

### Data analysis

Wind speed (U) data derived from the weather station were also averaged to get daytime, nighttime, and diel means. Canopy temperatures (T_c_) from Exp-II and Exp-III were averaged to determine mean daytime (06:00-18:00), nighttime (18:00-06:00), and diel temperatures. Data for reference and IRH-warmed plots were then expressed as deviations from ambient control (ΔT_c_). Data were averaged across all days within the experimental periods to determine mean deviation from control plot (ΔT_c_) for daytime (ΔT_c D_) and nighttime (ΔT_c N_). To investigate the dependence of achieved canopy warming on wind speed, we determined simple correlations of ΔT_c _with wind speed at different temporal scales.

## List of Abbreviations

Ck: Control/reference plot; FATE: Free-air Temperature Enhancement; HDT: High daytime temperature; HDNT: High daytime and nighttime temperature; HNT: High nighttime temperature; HT: High temperature with 12 heaters; IPCC: Intergovernmental Panel on Climate Change; IRH: Infrared Heater; IRT: Infrared Thermometer; OTC: Open-top chamber; PID: proportional-integrative derivative; Tc: Canopy Temperature; ΔTc: (IRH-warmed Tc) - (Reference Tc); ΔTc D: (Daytime IRH-warmed Tc) - (Reference Tc); ΔTc N: (Nighttime IRH-warmed Tc) - (Reference Tc); ΔTc DN: (Daytime and nighttime IRH-warmed Tc) - (Reference Tc).

## Competing interests

The authors declare that they have no competing interests and commercial names and details of equipments are for guideline only.

## Authors' contributions

MIAR and YFD envisioned the project and designed experiments. MIAR designed the instrumentation, BAK and MIAR designed datalogger program. MIAR, JQZ, SAUK, CY and GHL performed the experiments and analyzed the data. MIAR, SHW, ZHL and YFD developed modifications to the instrumentation and acquired funding. MIAR wrote the paper with contributions from all the authors. BAK and MIAR reviewed and edited later versions of draft. All authors have read and approved the final manuscript.
